# A pilot study of the clinical evidence for the methodology for prevention of oral mucositis during cancer chemotherapy by measuring salivary excretion of 5-fluorouracil

**DOI:** 10.1038/s41405-018-0008-2

**Published:** 2018-11-23

**Authors:** Akiko Kumagai, Shin Iijima, Takayuki Nomiya, Izuru Furuya, Yu Ohashi, Koichi Tsunoda, Kei Onodera, Naoko Tsunoda, Yuko Komatsu, Taifu Hirano

**Affiliations:** Iwate Medical University, Reconstructive Oral and Maxillofacial Surgery, Morioka, Iwate Japan

## Abstract

Objective to re-examine measures to prevent oral mucositis caused by drugs in head and neck cancer patients during cancer treatment by measuring salivary excretion of 5-fluorouracil. Saliva, blood, and urine were simultaneously collected from oral cancer patients and breast cancer patient at the point in time of before, during, and after the administration of 5-FU, then the 5-FU levels of the samples were quantitatively analysed using LC-MS/MS. In all patients, the 5-FU levels in saliva and serum peaked at 30 min to 3 h after the start of 5-FU treatment, and high levels were maintained throughout the administration of the drug. With regard to urinary 5-FU levels, they remained high from 3 to 120 h after the start of 5-FU treatment. After the completion of 5-FU treatment, even though it not appeared in the patients’ serum and urine promptly, 5-FU was detected in saliva at 12 h after the completion of 5-FU treatment in one oral cancer patient and at 48 h after the completion of 5-FU treatment in the breast cancer patient. It was suggested that the level of hydration after the completion of chemotherapy may be involved in the differences in 5-FU excretion.

## Introduction

To treat head and neck cancer, 5-fluorouracil (5-FU) is routinely used in combination with other anticancer agents or radiation, or as an oral anticancer agent for outpatients. However, adverse reactions include oral mucositis as a digestive symptom. The onset of oral mucositis deteriorates the general condition due to difficulty with ingestion, making continuous cancer treatment difficult in some patients. There are several hypotheses regarding the pathogenesis of mucositis.

Studies have demonstrated various mechanisms by which anticancer agents cause mucositis. Cytotoxic anticancer agents can cause damage to the basal cells by inducing cytokine and free radical release, leading to the inhibition of apoptosis and mucosal epithelium formation. Furthermore, bone marrow suppression caused by anticancer agents can lead to secondary mucositis as a result of an oral infection.^1^

Using inductively coupled plasma-mass spectrometry, we previously demonstrated that saliva from a patient with manic disorder contained a high concentration of one of the components of a drug, lithium carbonate, that the patient had been administered on a regular basis.^2^ The patient developed oral lichenoid drug reaction, but did not have any other lesions on the skin or in other areas of the body. We performed a drug-induced lymphocyte stimulation test and demonstrated that the patient was allergic to lithium carbonate. This finding suggested that the oral lesion was caused by lithium that was excreted with saliva and acted directly on the oral mucosa. Although many studies demonstrated that drugs are excreted into the saliva in addition to the blood and urine, few have examined the effect of drugs in saliva on the oral mucosa. Therefore, we performed a study to recognize the direct influence of a drug on the oral mucosa by showing the amount of 5-FU excreted from saliva as a numerical value.

The purpose of this study is to prevent oral mucositis and relieve the stress of head and neck cancer and to support treatment continuation by increasing motivation toward good oral care while undergoing cancer treatment based on measuring salivary 5-FU levels, furthermore, to continue investigation by the methods and results of the present research as a pilot study.

## Subjects and methods

The subjects were oral cancer patients receiving cancer chemotherapy with 5-FU during admission to our department and breast cancer patients receiving it at the outpatient clinic of the Department of Surgery of our hospital. Informed consent regarding sample collection was obtained from all subjects (*n* = 8), but four were excluded from the subjects because the continuous collection of saliva was considered difficult due to the exacerbation of digestive symptoms during the sample collection period. Finally, reference samples could be collected from three patients with oral cancer and one with breast cancer, total four subjects (Table 1). The chemotherapy protocols of each patient are shown in Table 2.

**Table 1:** Subjects

	Gender	Age	Region	Protocol
1	Male	49	Oropharyngeal	1
2	Male	62	Mandibular gingiva	1
3	Male	79	Tongue	1 (60% does)
4	Female	40	Breast	2

**Table 2:** Protocols of chemotherapy

Protocol 1 (inpatient)	Day 1	2	3	4	5	6
DTX 40 mg/m^2^ (i.a.)	↓					
CDDP 60 mg/m^2^/2 h (i.v.)		↓				
5-FU 600 mg/m^**2**^/24 h (i.v.)			↓	↓	↓	**↓**
Protocol 2 (outpatient)	Day 1					
EPI 100 mg/m^2^/5 min. (i.v.)	↓					
CPA 500 mg/m^2^/30 min. (i.v.)	↓					
5-FU 500 mg/m^**2**^/30 min. (i.v.)	↓					

*DXT* docetaxel hydrate, *CDDP* cisplatin, *5-FU* 5-fluorouracil, *EPI* epirubicin hydrochloride, *CPA* cyclophosphamide hydrate

Saliva, blood, and urine were simultaneously collected from three oral cancer patients, and saliva and blood were from one breast cancer patient at the point time of before, during, and after the administration of 5-FU. Saliva on stimulation obtained by chewing a cotton roll in the oral cavity for 2 min using a Salivette® cotton swab (Sarstedt K.K., Nümbrecht, Germany) was adopted as a sample. The saliva-containing cotton roll was inserted into a centrifuge tube and centrifuged at 3000 rpm for 10 min. The supernatant was collected and used for measurement. In addition, blood and urine were collected at the same time points as saliva collection as thoroughly as possible. After centrifugation, serum and urine were used for measurement. These samples were stored at –20 °C until measurement. 5-FU levels of the samples were quantitatively analysed using liquid chromatography-mass spectrometry (LC-MS/MS). The analysis was performed by the Foundation for Promotion of Material Science and Technology of Japan (Tokyo, Japan). For pretreatment, samples after liquid-liquid extraction with ethyl acetate were concentrated and redissolved in an LC mobile phase to be measured using Prominance UFLC system (Simadzu Co., Kyoto, Japan) and QTRAP® 4500 (AB Sciex Co. Ltd., Tokyo, Japan) with a Synergi™ 4 μm Fusion-RP column (50 mm × 2.0 mm, 4.0 μm, Shimadzu GLC Ltd. Tokyo, Japan) at 40 °C. Mobile phases A (95% 10 mM ammonium formate + 5% methanol) and B (5% 10 mM ammonium formate + 95% methanol) were employed for gradient elution. The samples were measured twice by the electrospray ionization method with transition of *Q*1/*Q*3: 129.0/42.1 to determine mean values as quantitative results. Based on the data obtained, we examined the changes in salivary 5-FU levels associated with the administration of 5-FU and compared the data with those for blood and urinary levels.

## Results

**Fig. 1:**
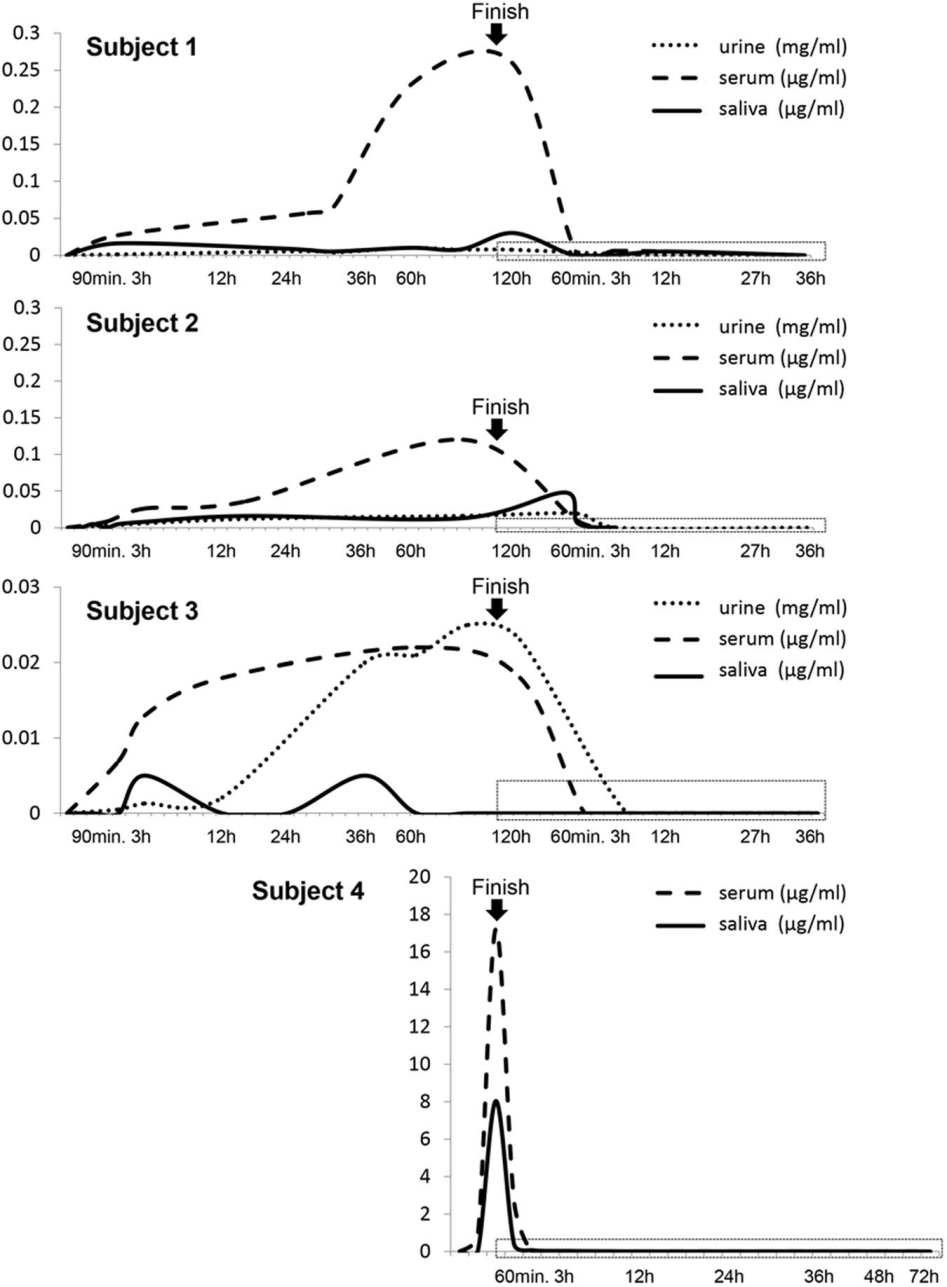
Overview of the levels of 5-FU of each sample (urine, serum, saliva) from starting point until after completion of cancer chemotherapy. The salivary and serum levels of 5-FU increased from 30 minutes to 3 hours after the start of 5-FU administration, and high levels were maintained throughout the administration of the drug. The patients' urinary 5-FU levels increased during 3 to 12 hours after the start of the 5-FU treatment. The dot squares of each graph show the area of Figure 2

Table 3 shows the 5-FU levels of each sample, urine, serum, and saliva, from the start of 5-FU treatment to after the end of administration. In all cases, the salivary and serum levels of 5-FU increased from 30 min to 3 h after the start of 5-FU administration, and high levels were maintained throughout the administration of the drug. With regard to the patients’ urinary 5-FU levels, they were increased from 3 to 12 h after the start of the 5-FU treatment (Fig. 1).

**Table 3:** Levels of 5-FU of each sample (urine, serum, saliva) during 5-FU treatment and after completion of administration

Subject number	Samples	Before	After start of 5-FU administration							After finish of 5-FU administration						
			30 min	90 min	3 h	12 h	36 h	60 h	120 h	30 min	60 min	3 h	12 h	36 h	48 h	72 h
1	Urine (mg/ml)	0		0.0011			0.0059	0.0100	0.0074				0	0	–	–
	Serum (μg/ml)	0		0.0270				0.2300	0.2600		0.0050	0.0060	0.0050	0	–	–
	Saliva (μg/ml)	0		0.0160				0.0100	0.0300	0.0050	0	0	0.0050	0	–	–
2	Urine (mg/ml)	0	0.0030			0.0120			0.0160		0.0190	0	0	0	–	–
	Serum (μg/ml)	0	0.0080		0.0260	0.0360			0.1200		0.0090	0	0	0	–	–
	Saliva (μg/ml)	0	0.0050	0.0050	0.0080	0.0160			0.0130	0.0480	0.0060	0	0	0	–	–
3	Urine (mg/ml)	0		0.0005	0.0013	0.0021	0.0200	0.0210	0.0230			0.0005	0	0	–	–
	Serum (μg/ml)	0		0.0070	0.0130	0.0180		0.0220	0.0180		0	0	0	0	–	–
	Saliva (μg/ml)	0	0	0	0.0050	0	0.0050	0	0	0	0	0	0	0	–	–
4	Serum (μg/ml)	0	17.2400	–	–	–	–	–	–	0	0	0	0	0	0	0
	Saliva (μg/ml)	0	8.0340	–	–	–	–	–	–	0.0700	0.0300	0.0150	0.0050	0.0050	0.0050	0

**Fig. 2:**
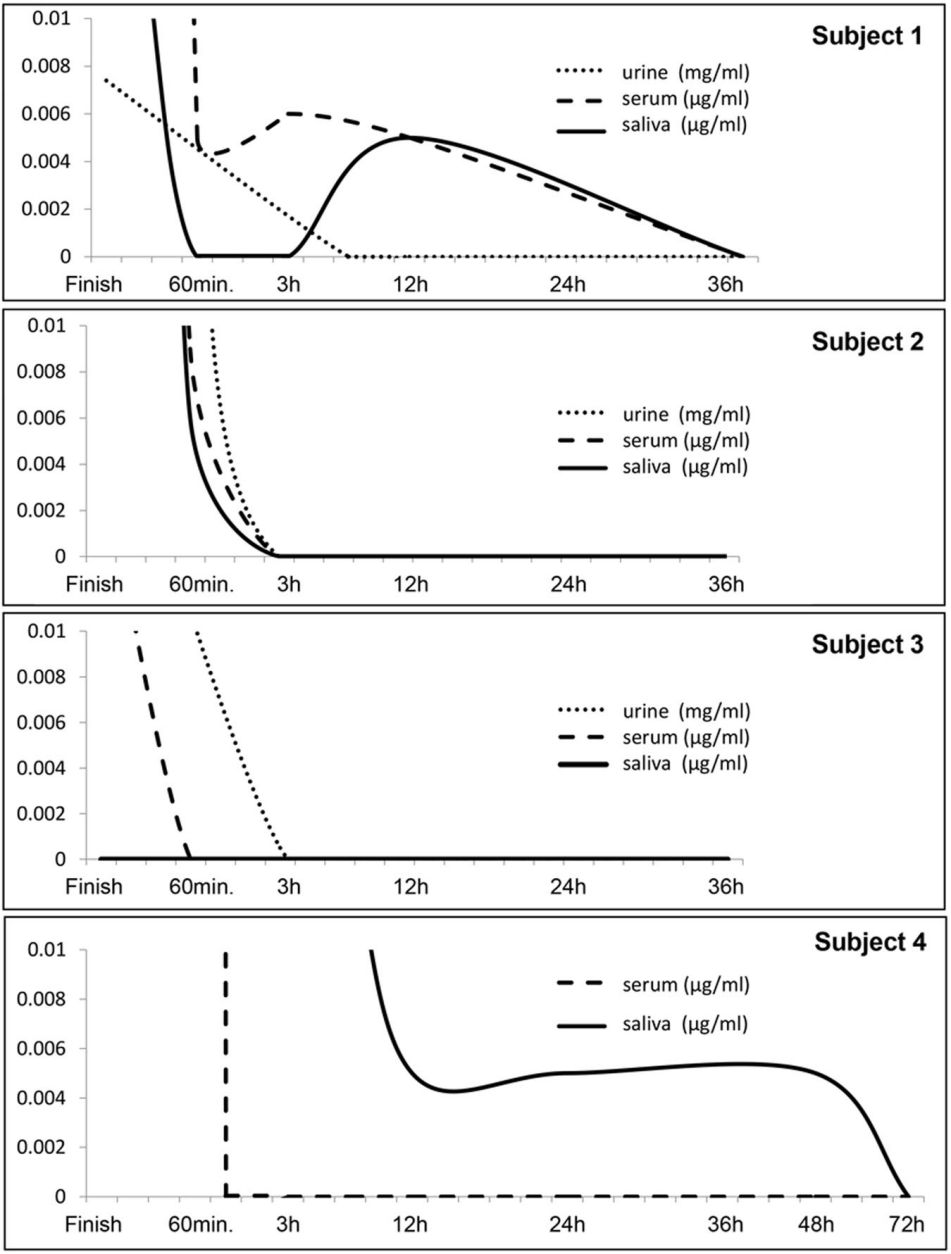
Levels of 5-FU of each sample (urine, serum, saliva) from finish point of cancer chemotherapy until no detection of 5-FU. It was detected in saliva ar 12 hours after the completion of the 5-FU treatment in subjects 1 and 4, respectively. In subject 4, 5-FU was detected in the saliva even after 48 hours even though it was not present in their sera at these time points.

Levels of 5-FU of all samples were decreased promptly at the end of 5-FU administration. Although the drug was detected only in the serum of subject 1 at 12 h after the completion of the 5-FU treatment, it was not detected in the other subjects’ sera at ≥3 h after the completion of the 5-FU treatment. It was also almost undetectable in the urine excreted 3 h after completion of 5-FU treatment. However, 5-FU was detected in saliva at 12 h after the completion of the 5-FU treatment in subjects 1 and 4, although its concentration was very low (<0.005 mg/ml). In subject 4, 5-FU was detected in the saliva even after 48 h even though it was not present in their sera at these time points (Fig. 2). There were no marked diurnal changes of 5-FU in saliva.

## Discussion

Oral mucositis related to a metabolic antagonist, 5-FU, may inhibit cellular metabolism, inducing oral mucosal injury. However, a recent study indicated that reactive oxygen species (ROS), produced in saliva, damaged basal cells in the oral mucosal epithelium, affecting the barrier function and contributing to the onset of anticancer drug–related stomatitis, suggesting the necessity of gargling with an ROS-scavenging agent.^3, 4^ Concerning 5-FU administration methods, continuous intravenous drip is commonly performed every day for patients with head and neck cancer. In this case, the oral mucosa may always be exposed to 5-FU contained in saliva, as demonstrated by the results of this study. In addition, there are diurnal changes in saliva excretion, suggesting the presence of hours during which a high concentration of 5-FU is excreted, which could not be clarified in this study. In patients with oral cancer, combined radiotherapy may markedly decrease saliva secretion, reducing self-purification. In addition, high concentrations of excreted 5-FU may remain in the oral cavity. Therefore, ROS levels may be saturated in the oral cavity of such patients.

There is a necessity to broaden the research to arrive at consistent results for the present study to advance to the next stage, so not only saliva but also mucosae must be a focus for the samples. The mechanisms of action on the oral mucosa should be elucidated from other views, such as cytology before, during, and after exposure of the mucosa by contact with saliva containing 5-FU. A five-phase chronological process has been proposed to explain mucositis, involving (1) cell death, (2) the generation of reactive oxygen species, (3) the activation of biological pathways, (4) ulceration, and (5) mucosalization. It has known that mucositis-like reactions do not occur immediately after mucosal contact with 5-FU-impregnated saliva, but after the end of apoptosis of the phase 2.^5, 6^ For further exploration of these findings, is the necessity of applying studies in animals in which biopsy is possible and to analyze the level of intracellular or interstitial impregnation of the drug.

A previous study reported that the saliva/plasma ratio of 5-FU was higher after high-dose administration or at a higher plasma concentration of 5-FU.^7, 8^ Therefore, gargling during 5-FU administration may be effective for preventing adverse reactions. Although many methods of using various types of mouthwash effectively have been reported, their preventive effects are clinically insufficient in most cases.^9, 10^ In addition, some types of mouthwash reportedly inhibit the antitumor effects of 5-FU; therefore, this study was significant for reviewing the selection of effective mouthwash or timing of gargling.

In saliva samples from two inpatients with oral cancer (subjects 2 and 3), 5-FU was not detected relatively soon after the completion of 5-FU treatment, whereas it was detected in saliva from an inpatient with oral cancer (subject 1) and an outpatient with breast cancer (subject 4) two days after the completion of 5-FU treatment. This might have been because pre- and post-5-FU administration parenteral fluid therapy made it possible for the former subjects to maintain a sufficient volume of circulating body fluid, thereby facilitating prompt 5-FU excretion. In fact, subjects 1 and 4 did not undergo parenteral fluid therapy after the administration of 5-FU, whereas subjects 2 and 3 continued to receive high-calorie parenteral therapy after the administration of 5-FU due to appetite loss. For outpatient care, hydration based on self-management at home is necessary after the completion of administration. This study suggests that hydration during and after anticancer-drug administration contributes to the prevention of oral mucositis.

Research on saliva-applying methods that replace blood concentration monitoring has been promoted by several organizations in the pharmaceutical field,^11–14^ but no study has aimed at the prevention of antitumor drug-induced oral mucositis. The results of this study indicate that it is necessary to employ oral care techniques, such as gargling, and to control circulating body fluids to prevent the 5-FU contained in saliva from having direct effects on the oral mucosa, suggesting the need for patient education during cancer treatment.

## Conclusions

This study was able to measure salivary 5-FU levels of the patients during 5-FU treatment. It was suggested that the level of hydration after the completion of chemotherapy may be involved in the differences in 5-FU excretion. As a result, we reconfirmed the importance of gargling during drug administration and that sufficient hydration during and after cancer chemotherapy is essential for the prevention of oral mucositis. The present study revealed the potential influence of a drug in saliva on the oral mucosa, and further studies on a larger number of subjects is needed to draw more firm conclusions.
